# ESCRT-0 marks an APPL1-independent transit route for EGFR between the cell surface and the EEA1-positive early endosome

**DOI:** 10.1242/jcs.161786

**Published:** 2015-02-15

**Authors:** Neftali Flores-Rodriguez, David A. Kenwright, Pei-Hua Chung, Andrew W. Harrison, Flavia Stefani, Thomas A. Waigh, Victoria J. Allan, Philip G. Woodman

**Affiliations:** 1 Faculty of Life Sciences, University of Manchester, Manchester M13 9PT, UK; 2 Children's Medical Research Institute, 214 Hawkesbury Road, Westmead, NSW 2145, Australia; 3 School of Physics and Astronomy, University of Manchester, Manchester M13 9PT, UK; 4 Photon Science Institute, University of Manchester, Manchester M13 9PT, UK

**Keywords:** EGFR, Hrs, ESCRT-I, EEA1, Particle-based colocalisation

## Abstract

Endosomal sorting complexes required for transport (ESCRT)-0 sorts ubiquitylated EGFR within the early endosome so that the receptor can be incorporated into intralumenal vesicles. An important question is whether ESCRT-0 acts solely upon EGFR that has already entered the vacuolar early endosome (characterised by the presence of EEA1) or engages EGFR within earlier compartments. Here, we employ a suite of software to determine the localisation of ESCRT-0 at subpixel resolution and to perform particle-based colocalisation analysis with other endocytic markers. We demonstrate that although some of the ESCRT-0 subunit Hrs (also known as HGS) colocalises with the vacuolar early endosome marker EEA1, most localises to a population of peripheral EEA1-negative endosomes that act as intermediates in transporting EGFR from the cell surface to more central early endosomes. The peripheral Hrs-labelled endosomes are distinct from APPL1-containing endosomes, but co-label with the novel endocytic adaptor SNX15. In contrast to ESCRT-0, ESCRT-I is recruited to EGF-containing endosomes at later times as they move to more a central position, whereas ESCRT-III is also recruited more gradually. RNA silencing experiments show that both ESCRT-0 and ESCRT-I are important for the transit of EGF to EEA1 endosomes.

## INTRODUCTION

The epidermal growth factor receptor (EGFR) pathway serves as an excellent model to study the dynamics of mitogenic receptor signalling and downregulation. Once activated, EGFR is ubiquitylated, rapidly internalised and transported to the early endosome (Sorkin and Goh, 2009). From here, although some EGFR is recycled, a major portion is sorted into intralumenal vesicles (ILVs) within the vacuolar region of the endosome, which develops into the multivesicular body (MVB) (Raiborg and Stenmark, 2009; Sorkin and Goh, 2009). This morphological alteration is accompanied by changes in the biochemical features and localisation of the endosome, which eventually converts into a late endosome and delivers EGFR directly to the lysosome (Foret et al., 2012; Rink et al., 2005). As EGFR passes through the endocytic pathway, it encounters many scaffolding complexes that collectively determine how it is sorted and define the pattern of EGFR-dependent signalling (Sigismund et al., 2012). Hence, the precise localisation of these complexes to particular endosomal compartments would contribute to our understanding of the spatial and temporal control of EGFR signalling.

Classical studies differentiated early (i.e. ‘sorting’) endosomes from late and recycling endosomes, based in part on their complement of Rab GTPases (Zerial and McBride, 2001). Early endosomes are enriched for Rab5, whereas recycling compartments contain Rab4 or Rab11 and late endosomes are Rab7 positive. More recent analysis has discovered that the early endosome corresponds to a broader profile of compartments, all enriched in Rab5 but distinguished from each other by their cellular distribution, kinetics of labelling with endocytosed cargo markers and unique complement of Rab5 effector proteins and phosphoinositides. For example, vacuolar early endosomes are located somewhat centrally and are rich in phosphatidylinositol-3-phosphate (PtdIns3*P*), a lipid generated by the PtdIns 3-kinase, VPS34 (vacuolar sorting protein 34) (Christoforidis et al., 1999). They contain several Rab5 effectors that also bind to PtdIns3*P*, including the FYVE (Fab1-YOTB-Vac1-EEA1) domain-containing proteins EEA1 (early endosome antigen 1) (Mu et al., 1995; Simonsen et al., 1998) and rabenosyn-5 (Nielsen et al., 2000).

Prior to being delivered to EEA1-positive endosomes, some EGFR might pass through a more peripheral population of Rab5-positive early endosomes. These small endosomes are enriched in APPL1 (adaptor protein, phosphotyrosine interaction, PH domain and leucine zipper containing 1), an adaptor protein important for Akt signalling and cell survival (Miaczynska et al., 2004; Schenck et al., 2008). Although APPL1 binds to Rab5, tyrosine-phosphorylated receptors and phosphoinositides, it does not display the same selective binding to PtdIns3*P* shown by EEA1 (Chial et al., 2008; Miaczynska et al., 2004). APPL1 also associates with endosomes containing nerve growth factor receptors (Fu et al., 2011; Lin et al., 2006; Varsano et al., 2006) and adiponectin receptor (Mao et al., 2006), suggesting that APPL1 endosomes perform a general role in regulating post-internalisation signalling processes. Over time, these signalling endosomes lose APPL1 and acquire EEA1 as they relocate to the cell centre (Zoncu et al., 2009), suggesting that the APPL1 endosome is a general intermediate in EGFR trafficking. This ‘maturation’ is not direct, but involves an intermediate early endosome that is enriched in the novel Rab5 effector WDFY2 (WD repeat and FYVE domain containing 2) (Zoncu et al., 2009), a protein that has also been implicated in early steps of transferrin receptor (TfR) uptake (Hayakawa et al., 2006). Interfering with the maturation pathway prolongs residency of EGFR in APPL1-positive endosomes and sustains EGFR-dependent APPL1 signalling (Zoncu et al., 2009).

Within the early endosome, ubiquitylated EGFR engages the endosomal sorting complex required for transport (ESCRT) pathway, a series of protein complexes that are believed to sort the receptor into ILVs (Hanson and Cashikar, 2012; Henne et al., 2011; Hurley and Hanson, 2010). ESCRT-0 is a heterodimeric complex of hepatocyte growth factor receptor substrate (Hrs, also known as HGS) and signal transducing adaptor molecule (STAM) (Ren et al., 2009) that acts early in the ESCRT pathway to sequester ubiquitylated cargo (Ren and Hurley, 2010). ESCRT-0 combines with ESCRT-I and ESCRT-II to initiate the invagination of the endosomal membrane (Wollert and Hurley, 2010), while a downstream complex, ESCRT-III, drives the formation of ILVs (Hanson and Cashikar, 2012; Henne et al., 2011; Hurley and Hanson, 2010; Wollert and Hurley, 2010). One crucial question is when and where the sequestration of EGFR by ESCRT-0 first occurs prior to ILV formation. Within vacuolar early endosomes, where ILV formation is initiated, activated EGFR is found in the ILVs and also on the limiting membrane, where it localises predominantly to domains that are enriched in clathrin and the ESCRT-0 subunit Hrs (Raiborg et al., 2001; Sachse et al., 2002), but depleted of EEA1 (Raiborg et al., 2001). These Hrs- and clathrin-enriched regions of the endosome also concentrate artificially ubiquitylated cargo (Raiborg et al., 2002). These data imply that ESCRT-0-dependent sorting occurs principally after cargo has reached the limiting membrane of vacuolar EEA1-positive endosomes, immediately prior to ILV formation. An alternative model argues that cargo separation begins at the plasma membrane, with degradative cargo being packaged selectively into a unique complement of endocytic vesicles that are highly motile and fuse directly with rapidly maturing late endosomes (Lakadamyali et al., 2006).

To address whether the initial interaction between EGFR and ESCRT-0 occurs within the EEA1-positive vacuolar endosome or in earlier compartments, we examined the localisation of the ESCRT-0 subunit Hrs relative to that of other endocytic markers using bespoke object-based colocalisation analysis software. We identify that a significant portion of ESCRT-0 encounters EGF in peripheral endosomes that lack APPL1 but instead label for the recently identified early endosome marker SNX15 (Danson et al., 2013). Downstream ESCRT complexes accumulate more steadily as EGF transits to larger centrally located early endosomes. Furthermore, the transit of EGF to EEA1-positive endosomes is ESCRT dependent.

## RESULTS

### Endogenous Hrs localises to peripheral compartments that are depleted of EEA1

**Fig. 1.**
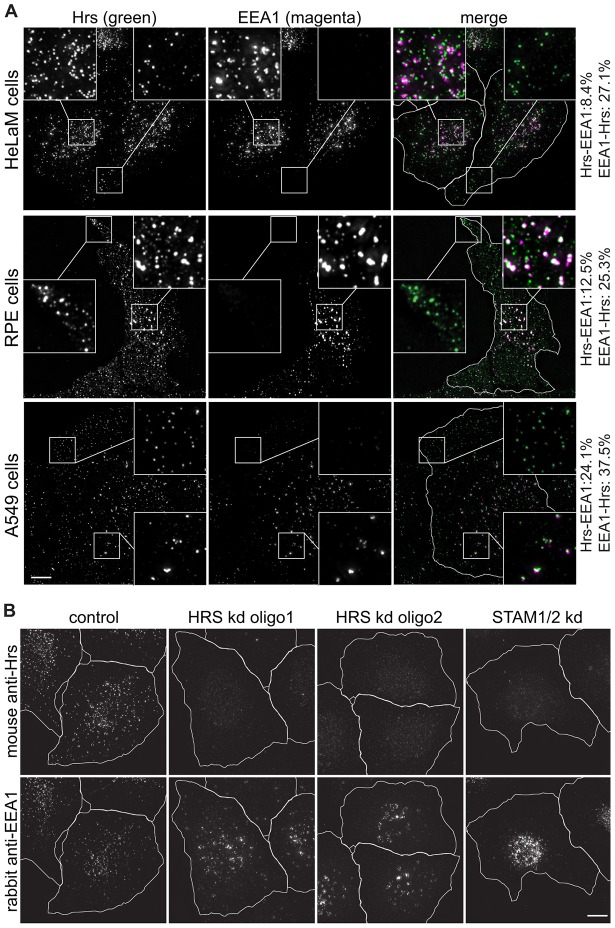
**Identification of peripheral Hrs-positive endosomes lacking EEA1.** (A) HeLaM (upper panel), RPE (centre panel) or A549 cells (lower panel) were co-stained with rabbit anti-Hrs and mouse anti-EEA1. (B) HeLaM cells were knocked down for ESCRT-0 components as indicated and stained with mouse anti-Hrs and rabbit anti-EEA1. The insets show a ×3 magnification of the indicated areas. Values show net percentages (i.e. scrambled values subtracted) of particles labelled by marker 1 that are also labelled by marker 2. kd, knockdown. Cell outlines are shown in white. Scale bars: 10 µm.

The distributions of endogenous ESCRT-0 and EEA1 were first examined in HeLaM cells by immunofluorescence microscopy, using a rabbit polyclonal antibody against Hrs (see supplementary material Fig. S1A for its specificity by western blotting) and a well-characterised monoclonal antibody against EEA1. As expected, there was considerable overlap between Hrs and EEA1 in more central areas of the cell (Fig. 1A, upper panels, insets), in line with previous data (Raiborg et al., 2001). However, many Hrs-positive structures, particularly those situated towards the cell periphery, appeared to lack, or labelled very weakly for, EEA1 (Fig. 1A; upper panels, insets). This differential localisation of ESCRT-0 and EEA1 was not restricted to HeLaM cells, because strong colocalisation of Hrs and EEA1 was also limited to more central endosomes in RPE (Fig. 1A, centre panels) and A549 (Fig. 1A, lower panels) cells. Notably, in RPE cells ‘Hrs-only’ endosomes were abundant in the extended peripheral regions. We also labelled cells with a different combination of antibodies [mouse anti-Hrs (see supplementary material Fig. S1A for blot) and rabbit anti-EEA1]. These also showed partially overlapping staining, with more Hrs in the cell periphery (supplementary material Fig. S1B). The two antibodies against Hrs gave virtually identical labelling patterns when combined, as did the two anti-EEA1 antibodies (supplementary material Fig. S1C,D). Hence, the different observed localisation of ESCRT-0 and EEA1 cannot be explained simply by limited accessibility of epitopes within certain cellular locations or by non-specific labelling.

The specificity of the anti-Hrs staining was confirmed by knockdown of Hrs using two independent small interfering (si)RNAs, which caused EEA1 to cluster centrally and essentially abolished vesicular Hrs labelling by both antibodies (Fig. 1B; supplementary material Fig. S1E). Because we have not identified any antibodies against the other ESCRT-0 subunit, STAM, that work well for immunofluorescence, it remains formally possible that we localised a pool of Hrs that functions independently of ESCRT-0. However, this is highly unlikely, given that essentially all cellular pools of Hrs and STAM are coassembled into ESCRT-0 (Kobayashi et al., 2005; Ren et al., 2009). Indeed, combined knockdown of STAM1 and STAM2 isoforms also abolished vesicular Hrs labelling (Fig. 1B; supplementary material Fig. S1E). EEA1 clustering was also observed upon STAM knockdown, although the pattern of EEA1 was somewhat different from that observed upon Hrs knockdown.

To provide more quantitative information about the extent of colocalisation between EEA1 and Hrs, we developed a colocalisation algorithm. Common methods for analysing colocalisation, such as the Pearson's and Spearman's correlation coefficients, rely on intensity-based analysis, whereby the intensity of one colour channel is compared with another for each pixel within the image, in order to determine how well the overall fluorescence patterns are matched. An alternative method is object-based colocalisation (Bolte and Cordelières, 2006). Here, the precise localisation of markers is first obtained in each channel. The localisation maps are then compared, and if particles from each channel are within a threshold distance they are judged to label the same compartment. One advantage of object-based colocalisation compared to intensity-based analysis is that it provides an accurate measure of the number of compartments on which fluorescent labels coincide. An object-based colocalisation methodology has been used recently as the basis to determine the dynein and kinesin motor copy number on vesicles containing amyloid precursor protein (Szpankowski et al., 2012).

First, the location of fluorescent structures labelled in each channel was obtained with subpixel resolution. For this, we used the particle-tracking programme, PolyParticleTracker. This identifies the centre of a particle by fitting the diffraction limited fluorescence intensity using a polynomial-fit, Gaussian-weight algorithm, and it can track fluorescent particles with an error of <20 nm (Rogers et al., 2010; Rogers et al., 2007). Using this software, we generated maps of particle centre locations in each channel. These maps were overlaid, and every particle in each channel was scored for whether a corresponding particle was also observed in the other channel(s) after applying a distance threshold (215 nm, corresponding to two pixels). Hence, fluorescence punctae that are separated by more than this threshold are likely to reside within distinct endosomes, although a small number might reside in well-separated domains of larger endosomes located towards the cell centre. The colocalisation software, termed COLOCAL, is available online.

To provide standards against which to measure the degree of colocalisation between Hrs and EEA1, the colocalisation of rabbit and mouse anti-Hrs antibodies, which by eye gave extremely similar staining patterns (supplementary material Fig. S1C), was quantified. The antibodies labelled approximately the same number of particles, and the degree of measured colocalisation between channels was very high, although not absolute (supplementary material Table S1A). The failure to reach 100% measured colocalisation might be accounted for, at least in part, by slight differences in the sensitivity and background labelling of the two antibodies, which in turn can generate false-positive or false-negative structures close to the signal∶noise threshold. Rabbit and mouse anti-EEA1 also colocalised extensively (supplementary material Fig. S1D), and a high but not absolute degree of colocalisation between these markers was measured (supplementary material Table S1A). In line with our qualitative observations, the object-based colocalisation software demonstrated that in HeLaM, A549 and RPE cells, relatively few Hrs particles also possessed significant amounts of EEA1, although somewhat more EEA1-labelled structures also contained Hrs (supplementary material Table S1A).

### Hrs localises to SNX15-positive but not APPL1-positive endosomes

**Fig. 2.**
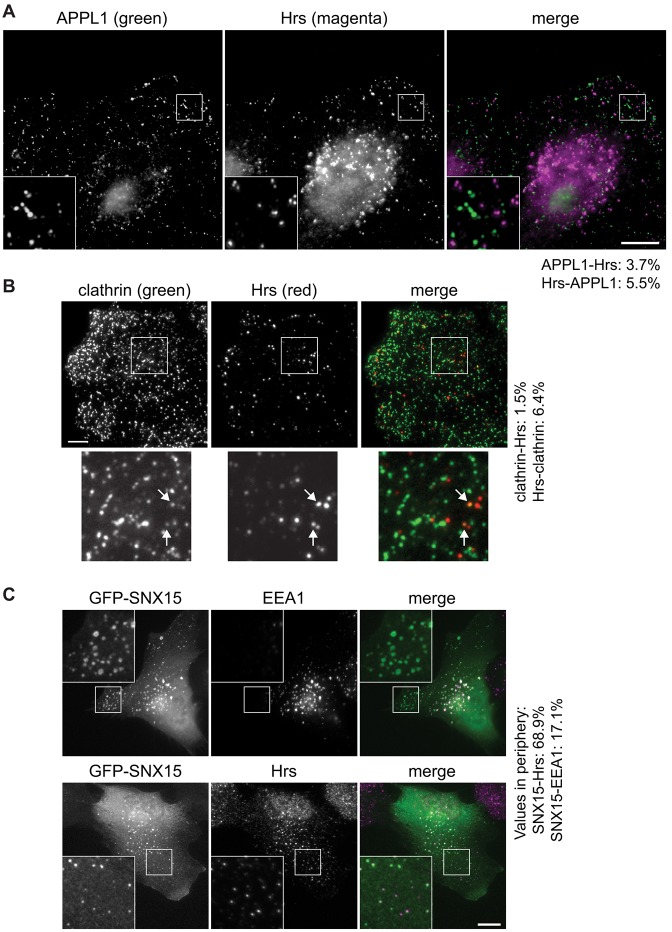
**Localisation of peripheral Hrs to SNX15 but not APPL1 endosomes.** (A) HeLaM cells were co-stained for APPL1 and Hrs, and were examined by wide-field microscopy. (B) HeLaM cells were stained for clathrin and Hrs and imaged by TIRF microscopy. Arrows show examples of structures containing both Hrs and clathrin. (C) HeLaM cells were transfected with GFP–SNX15, fixed and stained for EEA1 and Hrs and examined by wide-field microscopy. The insets show a ×3 magnification of the indicated areas. Values show net percentages (i.e. scrambled values subtracted) of particles labelled by marker 1 that are also labelled by marker 2. Scale bars: 10 µm.

Because much of the Hrs localises to peripheral early endosomes containing very little EEA1, we asked whether these endosomes contained APPL1, a marker for endosomes that are rapidly encountered by EGFR upon its internalisation (Miaczynska et al., 2004), and which might serve as intermediate compartments en route to EEA1-positive endosomes (Zoncu et al., 2009). However, although Hrs and APPL1 showed broadly similar cellular distributions, there was virtually no overlap between the two markers. This was particularly evident when the peripheral regions of cells were imaged at higher magnification (Fig. 2A), and was confirmed by quantitative analysis (supplementary material Table S1B).

It was possible that a significant number of the peripheral Hrs-only endosomes were, in fact, clathrin-coated pits. We therefore performed total internal reflection fluorescence microscopy (TIRFM) to detect endogenous clathrin and Hrs at, or just below, the plasma membrane. The staining patterns for the two proteins were highly dissimilar (Fig. 2B). Hence, although Hrs and clathrin punctae overlapped occasionally in regions of very high clathrin density, close inspection suggested that their structures were distinct and most likely overlapped by chance (data not shown). A small number of bright Hrs punctae did label weakly for clathrin (Fig. 2B, arrows in inset) and might represent incompletely uncoated vesicles or nascent endosomes that contain a small amount of clathrin. The limited colocalisation between Hrs and clathrin was confirmed by quantitative analysis (supplementary material Table S1C). In summary, although we cannot be sure that ESCRT-0 never associates with coated pits, any such event must be rare.

SNX15 has recently been described as a novel effector of early endosomal trafficking that associates with EEA1-positive endosomes, but also with clathrin-coated intermediates and with peripheral endocytic vesicles that form upstream of EEA1 endosomes and are distinct from APPL1 endosomes (Danson et al., 2013). We therefore tested whether the peripheral Hrs endosomes corresponded to those containing SNX15. Because antibodies that recognise endogenous SNX15 are not available, we transfected cells with low levels of GFP–SNX15 and stained cells for endogenous Hrs and EEA1. As reported previously (Danson et al., 2013), GFP–SNX15 only partially colocalised with EEA1, with many peripheral GFP–SNX15 structures lacking EEA1 staining (Fig. 2C, upper panel). In contrast, the colocalisation of GFP–SNX15 and Hrs was very high, particularly in more peripheral regions (Fig. 2C, lower panel). Quantitative analysis showed that in these regions nearly all SNX15-positive endosomes contained Hrs, with the percentage measured as Hrs positive (68.8%) being very close to that measured for mouse anti-Hrs versus rabbit anti-Hrs (supplementary material Table S1D). Rather fewer Hrs endosomes also contained detectable levels of SNX15 (Fig. 2C, lower panel; supplementary material Table S1D), although the relatively high cytoplasmic GFP–SNX15 content meant that low concentrations of vesicle-associated GFP–SNX15 were unlikely to be detected. Hence, we cannot establish definitively whether or not a population of peripheral Hrs-positive, SNX15-negative endosomes also exists. Few EEA1-positive endosomes were found at the cell periphery, such that only a small proportion (17.1%) of peripheral SNX15-positive endosomes labelled with EEA1 (supplementary material Table S1D).

### Hrs endosomes are a principal route of EGF uptake in RPE cells

**Fig. 3.**
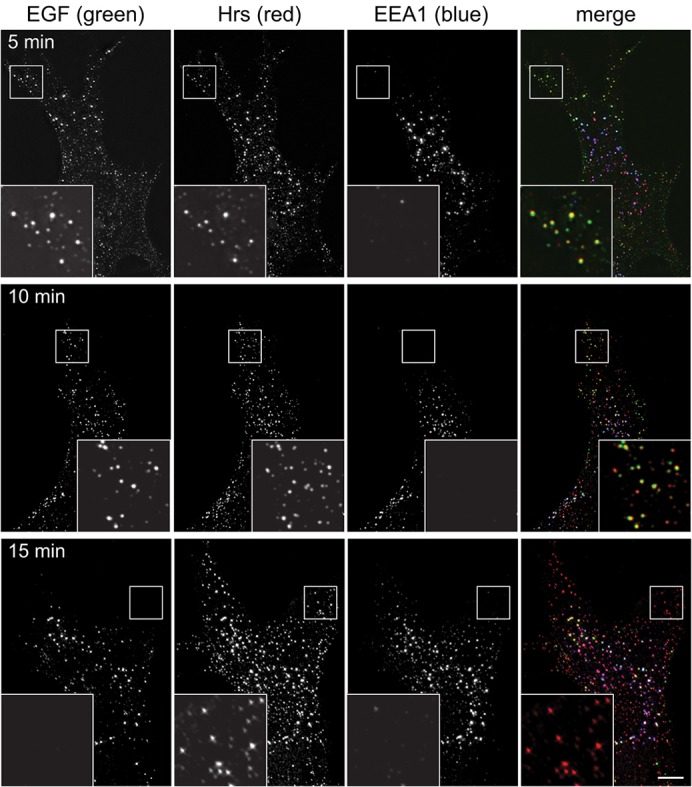
**EGF passes through peripheral Hrs endosomes prior to labelling EEA1 endosomes.** RPE cells were serum starved, then pulsed for 2 min with fluorescent EGF, then chased for a further 3 min (upper row), 8 min (middle row) or 13 min (lower row) before fixing and labelling with antibodies against Hrs or EEA1. The insets show a ×3 magnification of the indicated areas. Scale bar: 10 µm.

The presence of ESCRT-0 in a population of endosomes more peripheral than those positive for EEA1 suggested that at least some EGF–EGFR complex first encounters ESCRT-0 before it reaches larger vacuolar endosomes. To address the transit of EGF through these peripheral endosomes, experiments were performed in RPE cells that had been pulsed with fluorescent EGF for 2 min and chased for different lengths of time before fixation. After 5 min of total labelling, extensive colocalisation of EGF with Hrs was seen in the cell periphery (Fig. 3, upper panels). EGF also colocalised with Hrs in more central regions, in endosomes that also labelled strongly for EEA1. Both central and peripheral endosomes remained labelled for EGF after a 10-min incubation (Fig. 3, middle panels). However, by 15 min, EGF signal over the peripheral Hrs-positive EEA1-negative endosomes was profoundly diminished, and EGF labelling was confined to centrally located EEA1-positive endosomes that also contained significant amounts of Hrs (Fig. 3, lower panels). Hence, EGF transits peripheral Hrs-only endosomes earlier than central EEA1- and Hrs-positive endosomes.

**Fig. 4.**
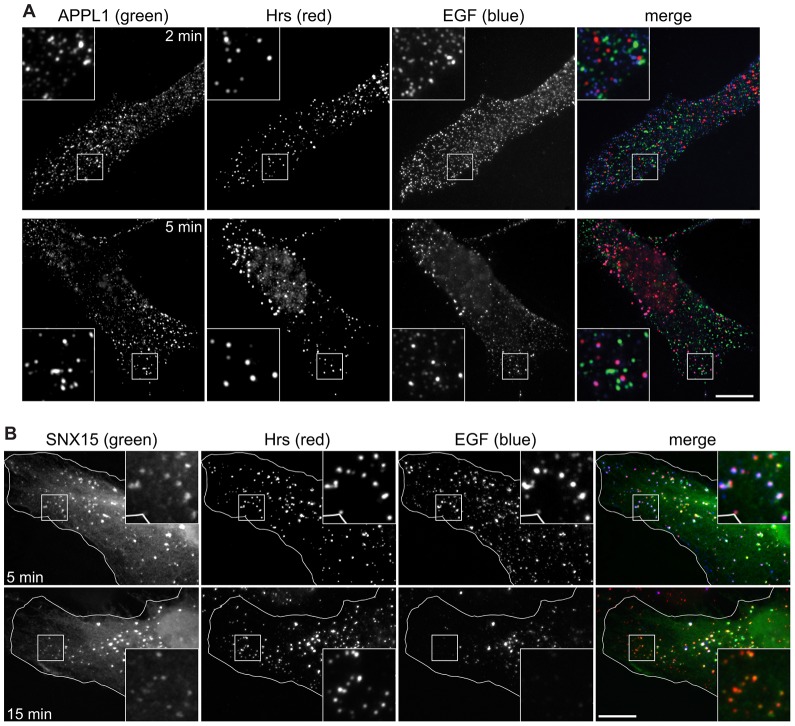
**EGF labels distinct Hrs/SNX15-positive and APPL1-positive endosomes.** Untransfected RPE cells (A) or RPE cells transfected with GFP–SNX15 (B) were serum starved and then pulse-chased with fluorescent EGF for the indicated total times and labelled for immunofluorescence. Cell outlines are shown in white. The insets show a ×3 magnification of the indicated areas. Scale bars: 10 µm.

Previous studies (Zoncu et al., 2009) have identified APPL1-positive endosomes as a principal station for EGF en route to vacuolar endosomes. To compare directly the relative amounts of internalised EGF passing through Hrs- or APPL1-positive endosomes, colocalisation analysis was carried out on images of EGF-stimulated cells. In RPE cells, both APPL1- and Hrs-positive structures were labelled with fluorescent EGF within 2 minutes of EGF uptake, although many EGF punctae were not yet labelled for either marker (Fig. 4A, upper panels) and might correspond to clathrin-coated pits or vesicles. Within 5 min, more EGF punctae labelled for Hrs, and few for APPL1 (Fig. 4A, lower panels). Significantly, at both time-points, more EGF localised to punctae containing Hrs than to those containing APPL1 {39.5% labelled with Hrs compared to 23.7% with APPL1 at 2 min [*n* = 1576; colocalisation threshold = 193 nm (3 pixels)], and 48.5% compared to 13.2% at 5 min (*n* = 730)}.

In HeLa cells only a fraction of EGF localises to APPL1 endosomes (Miaczynska et al., 2004) after a short uptake period. In COS7 cells, in which both APPL1 and EGFR are concentrated at the cell edge, the APPL1 endosome has been reported as a particularly significant uptake compartment for EGFR en route to later endocytic compartments (Zoncu et al., 2009). We therefore also compared Hrs, APPL1 and EGF distribution in these cells. After a 2-min pulse with fluorescent EGF, virtually all the EGF remained at or very close to the cell surface (data not shown). However, after a further 3-min chase, a significant portion of EGF had reached compartments that labelled either with APPL1 or Hrs, although many small structures labelling faintly for EGF still lacked either of these markers (supplementary material Fig. S2A). APPL1 was concentrated at the cell edge, mainly in small endosomes, whereas Hrs distribution was not as polarised. There was virtually no overlap between APPL1 and Hrs (supplementary material Fig. S2A). Several APPL1 endosomes at the extreme periphery contained EGF (supplementary material Fig. S2A, insets, closed arrows for examples), in line with previous reports (Zoncu et al., 2009), although many Hrs-positive endosomes also contained EGF (supplementary material Fig. S2). Quantification showed that, at this time-point, the proportion of these peripheral structures labelled lightly for EGF structures that also contained APPL1 was 22.8%; *n* = 5841; 13 cells, 2 experiments). Only 12.1% of these peripheral endosomes contained Hrs (although even in COS7 cells the Hrs-positive endosomes most likely accounted for a greater proportion of total EGF, given that Hrs endosomes were generally brighter than APPL1 endosomes for EGF; supplementary material Fig. S2A). In summary, although APPL1 endosomes might represent a significant route for entry of EGF in some cell types including COS7 (Zoncu et al., 2009), in other cells EGF rapidly and preferentially enters a separate population of peripheral endocytic vesicles that contain ESCRT-0.

Because SNX15 has been identified as an entry route to the endosomal system for EGF (Danson et al., 2013), we also compared SNX15 and Hrs labelling during a pulse of fluorescent EGF. As expected, SNX15- and Hrs-labelled structures in the cell periphery were accessed by EGF within 5 min of uptake, but were diminished for EGF labelling after 15 min as the EGF moved to more central endosomes (Fig. 4B). Quantification showed that, at 5 min of uptake, 89.9% of EGF-positive endosomes in the cell periphery that contained GFP–SNX15 also labelled for Hrs (210 endosomes; 6 cells; 2 experiments). Hence, ESCRT-0 and SNX15 populate the same endosomes that are accessed by EGF.

### Downstream ESCRTs are recruited later than ESCRT-0, as EGF-containing endosomes mature and acquire EEA1

**Fig. 5.**
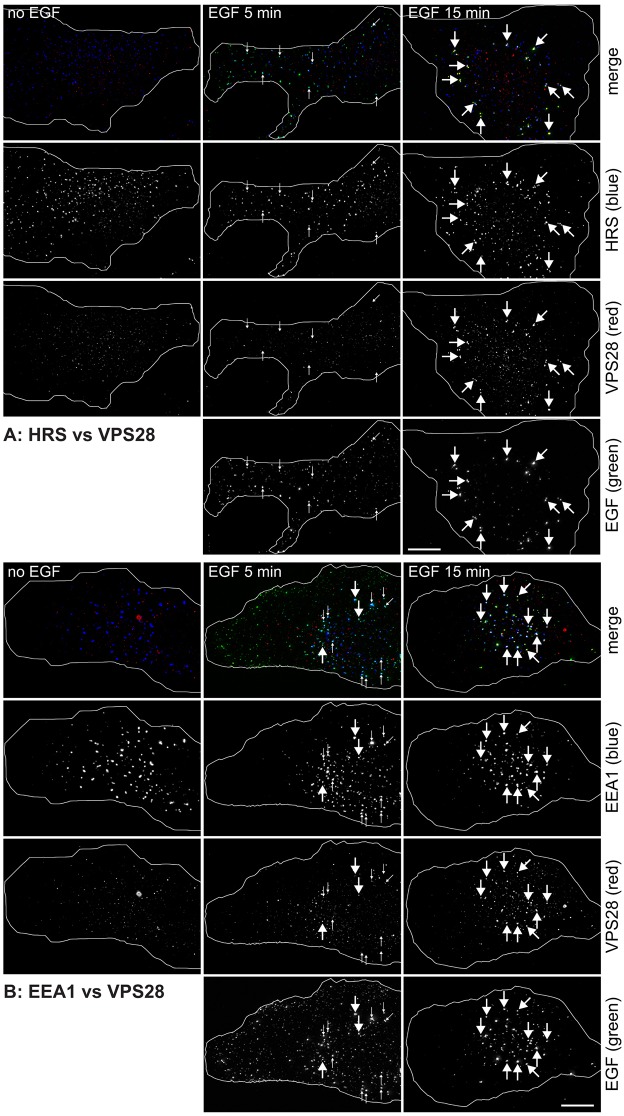
**Localisation of ESCRT-I to early endosomes.** RPE cells were serum starved and pulse-chased with fluorescent EGF for the indicated total times, permeabilised with saponin and labelled for VPS28 and Hrs (A) or EEA1 (B). Small arrows show examples of endosomes containing EGF and Hrs or EEA1 but lacking VPS28 labelling. Large arrows highlight example endosomes containing all three markers. Cell outlines are shown in white. Scale bars: 10 µm.

One question about the ESCRT-0 that localises to peripheral endosomes is whether this reflects the early initiation of intralumenal vesicle formation or whether ESCRT-0 might be required for EGFR trafficking in advance of the other components of the ESCRT pathway. We therefore examined the localisation of endogenous ESCRT-I and ESCRT-III complexes. ESCRT-I consists of four proteins – tumour susceptibility gene 101 (TSG101), vacuolar protein sorting 28 (VPS28), VPS37 and MVB protein of 12 kDa (MVB12) (Hanson and Cashikar, 2012). There are several variants of VPS37 (Bache et al., 2004; Eastman et al., 2005; Stuchell et al., 2004) and also of MVB12 (Morita et al., 2007; Tsunematsu et al., 2010). One of the MVB12 variants, ubiquitin-associated protein 1 (UBAP1), plays a crucial role in EGFR trafficking (Stefani et al., 2011). The other MVB12 variants might also be important for MVB biogenesis, although their roles are less well defined. Although there are many antibodies to ESCRT-I available, we have found only one, a previously characterised antibody against VPS28 (Bishop et al., 2002), which works reliably to detect the endogenous complex by immunofluorescence, and the endosomal pool of ESCRT-I is best visualised by permeabilising cells with saponin just prior to fixation to reduce cytoplasmic labelling. Under these conditions, in serum-starved RPE cells, the VPS28 antibody produced background labelling (Fig. 5A,B). Importantly, no VPS28 colocalised with either Hrs (Fig. 5A) or with EEA1 (Fig. 5B) prior to the addition of EGF. After 5 min of EGF internalisation, VPS28 staining remained barely detectable on Hrs or EEA1 endosomes, including those labelled for EGF (Fig. 5A,B). However, by 15 min, strong VPS28 labelling was found to colocalise with EGF (Fig. 5; supplementary material Table S1E for values at 15 min), often together with both Hrs (Fig. 5A) and EEA1 (Fig. 5B). To demonstrate the specificity of anti-VPS28 labelling, RNA silencing of TSG101 was performed in HeLaM cells using previously established conditions for siRNA (Bishop et al., 2002), because depletion of TSG101 causes almost complete loss of all ESCRT-I subunits including VPS28 (Stefani et al., 2011). No VPS28 labelling over EGF-containing endosomes was observed in cells depleted of TSG101 (supplementary material Fig. S2B) (note also that only a portion of EGF had reached EEA1 endosomes in these cells; see below). In summary, the association of ESCRT-I with early endosomes is absolutely dependent on the presence of cargo and occurs later than that of ESCRT-0.

**Fig. 6.**
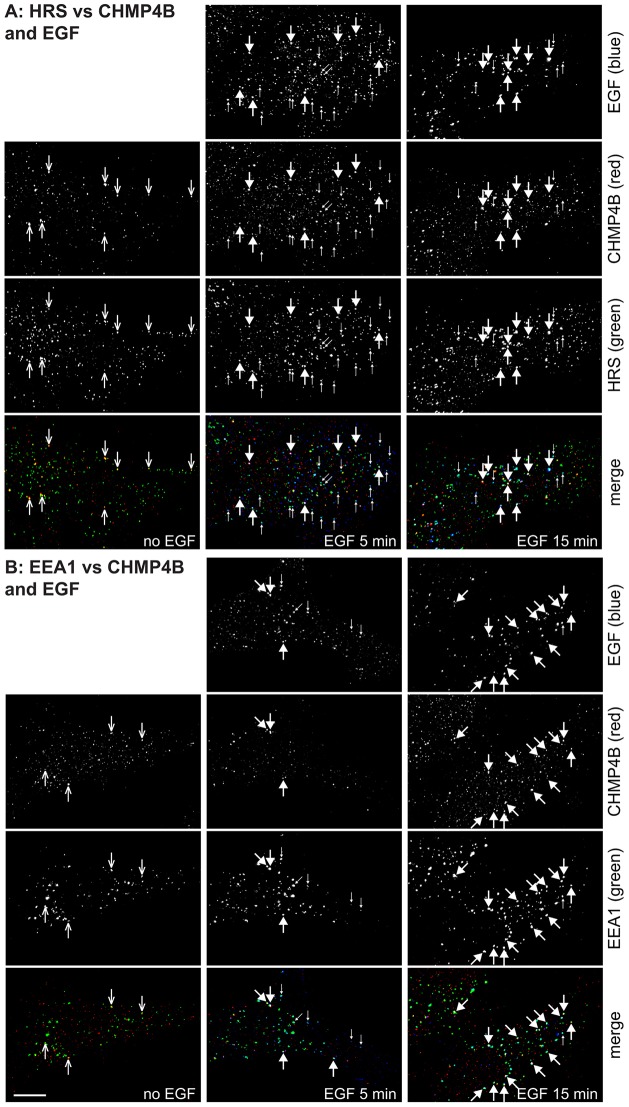
**Localisation of ESCRT-III to early endosomes.** RPE cells were serum starved and pulse-chased with fluorescent EGF for the indicated total times, permeabilised with saponin and labelled for CHMP4B and Hrs (A) or EEA1 (B). Arrows with open arrowheads show examples of CHMP4B in unstimulated cells colocalising with Hrs or EEA1. Small arrows show examples of endosomes containing EGF and Hrs or EEA1 but lacking CHMP4B labelling. Large arrows highlight example endosomes containing all three markers. Scale bar: 10 µm.

To visualise ESCRT-III, we used an antibody against CHMP4B that recognises CHMP4B and that does not crossreact with any other ESCRT-III subunit (Ali et al., 2013). As expected, CHMP4B staining was largely cytosolic, although some bright punctae were observed (supplementary material Fig. S3A). In cells lacking the ATPase VPS4, in which ESCRT components are locked on endosomes (Babst et al., 1998; Bishop and Woodman, 2001), the antibody gave very bright staining, much of which colocalised with EEA1 (supplementary material Fig. S3A). Hence, this antibody is an excellent marker for CHMP4B by immunofluorescence. The labelling pattern for endogenous CHMP4B versus EGF and other endocytic markers, which has not been examined before, was complex. In serum-starved RPE cells, treated with saponin prior to fixation to reduce the cytoplasmic label, CHMP4B was found in small bright punctae, a few of which colocalised with either Hrs or EEA1 (Fig. 6A,B; open arrows for examples). After 5 min of EGF internalisation, this pattern had not changed substantially, with some EGF endosomes containing CHMP4B as well as either Hrs and EEA1 (Fig. 6A,B; large arrows), although it was notable that many endosomes that were positive for Hrs and EGF lacked detectable CHMP4B labelling (Fig. 6A; small arrows). By 15 min, many (although by no means all) EGF endosomes had detectable levels of CHMP4B labelling, coinciding with Hrs or EEA1 labelling. Hence, although some CHMP4B is endosome-associated even in the absence of cargo, CHMP4B labelling on endosomes increases gradually during the first 15 min of EGF uptake. However, because some EGF-containing endosomes lack CHMP4B labelling, the association of CHMP4B might be rather transient. Similar findings were observed in HeLaM cells, in which CHMP4B colocalisation with fluorescent EGF peaked at 15 after uptake but remained incomplete (supplementary material Fig. S3B).

### ESCRTs are important for delivery of EGF between the cell surface and EEA1 endosomes

**Fig. 7.**
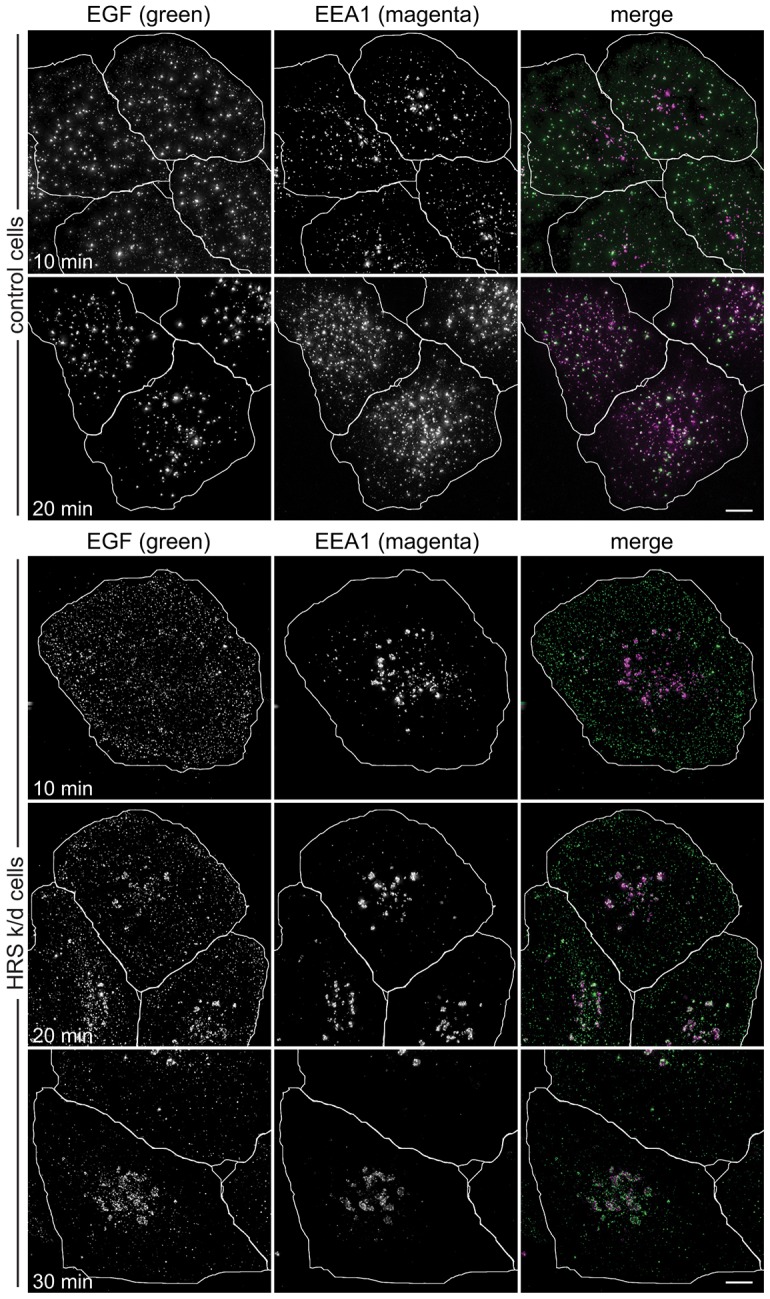
**ESCRT-0 is required for EGF transit to EEA1 endosomes.** HeLaM cells transfected with control siRNA or Hrs siRNA oligo 1 were pulse-chased with fluorescent EGF for the indicated total times, fixed for immunofluorescence and labelled with anti-EEA1. Cell outlines are shown in white. k/d, knockdown. Scale bars: 10 µm.

**Fig. 8.**
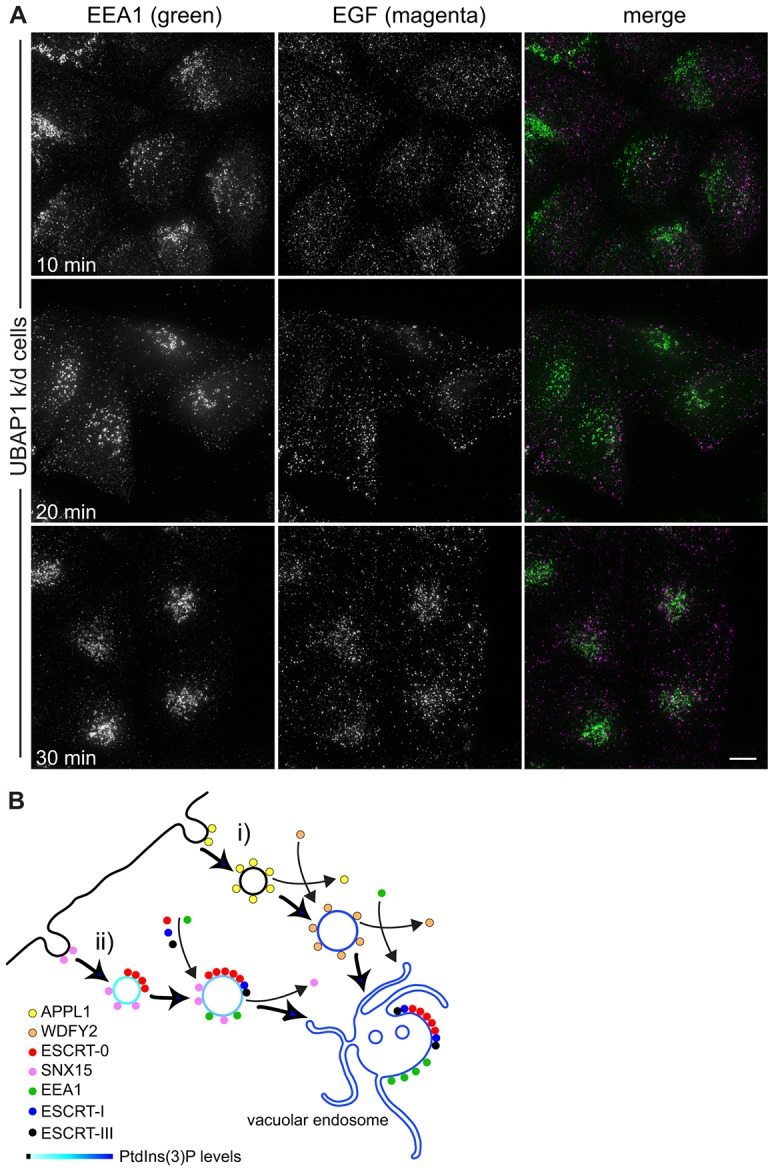
**ESCRTs and the organisation of the early endocytic pathway.** (A) HeLaM cells transfected with UBAP1 siRNA oligo 1 were pulse-chased with fluorescent EGF for the indicated total times and fixed for immunofluorescence. Scale bar: 10 µm. (B) EGFR can enter the endocytic system through at least two clathrin-mediated pathways. (i) APPL1 associates with clathrin pits and peripheral vesicles, then is replaced by WDFY2 as PtdIns3*P* is generated. WDFY2 in turn is replaced by EEA1 (see Zoncu et al., 2009). (ii) SNX15 and ESCRT-0 bind to a separate population of early endocytic intermediates. Generation of increasing levels of PtdIns3*P* as the vesicle develops, combined with increasing ubiquitylation of EGFR, might recruit more ESCRT-0, and ESCRT-I and ESCRT-III are also recruited alongside EEA1 as the endosome matures. ESCRT-0, but not SNX15 (Danson et al., 2013), enters clathrin-rich domains on the vacuolar endosome.

These data show that ESCRT-0 associates rapidly with EGF-containing early endosomes, whereas ESCRT-I associates somewhat later, as these endosomes mature as judged by recruitment of EEA1. We therefore tested whether ESCRT-0 and ESCRT-I have a role during early endosome maturation in addition to their known role in ILV formation. In control HeLaM cells, much EGF had reached central EEA1-positive endosomes after 10 min. This translocation of EGF was essentially complete by 20 min (Fig. 7, upper panels), and many EGF-positive endosomes still labelled for EEA1 after 30 min (data not shown). In contrast, in cells lacking Hrs, after 10 min, EGF had entered into very small and peripheral structures. Its translocation to central EEA1-positive structures, which were clustered, was much slower than in control cells, and was only partially achieved by 30 min (Fig. 7, lower panels). Similar findings were seen in cells treated with an independent Hrs siRNA (supplementary material Fig. S4A). Many of the peripheral EGF-labelled structures detected after 10 min of uptake in Hrs-depleted cells were internal, rather than located on the cell surface, because they were labelled with an antibody that detects the lumenal domain of EGFR only in detergent-permeabilised cells (supplementary material Fig. S4B). Hence, ESCRT-0 is important for the movement of EGF from small peripheral endosomes to larger centrally located structures. Knockdown of the ESCRT-I subunit UBAP1 also substantially delayed transfer of EGF from peripheral structures to centrally located EEA1-positive endosomes (Fig. 8; supplementary material Fig. S4C,D), and loss of the central ESCRT-I subunit, TSG101, generated a similar phenotype (supplementary material Fig. S2B). Therefore, in keeping with their localisation to nascent or maturing early endosomes, ESCRT-0 and ESCRT-I are important for this maturation process to continue efficiently. This reveals a novel role for ESCRTs upstream of their known activity in ILV generation.

## DISCUSSION

In this report, we describe the localisation of endogenous ESCRT complexes within the early endocytic system, with particular focus on ESCRT-0, the complex that acts at the apex of the ESCRT pathway. We demonstrate that a significant portion of membrane-associated ESCRT-0 localises to small endosomes distributed in the cell periphery. These endosomes either lack EEA1 altogether or contain low levels of EEA1 that are not readily detected by immunofluorescence microscopy. As reported previously (Raiborg et al., 2001), ESCRT-0 also localises to regions within more central vacuolar endosomes enriched for EEA1, which are likely to correspond to MVBs in which EGFR is being sorted into ILVs. The peripheral endosomes enriched for ESCRT-0 co-label with SNX15, which is necessary for the entry of both EGFR and TfR into the endosomal system (Danson et al., 2013). Furthermore, many are accessed by EGFR soon after the receptor is activated by fluorescent EGF, in line with the rapid association of ESCRT-0 with EGFR that has been detected biochemically (Ali et al., 2013; Umebayashi et al., 2008). Peripheral ESCRT-0 endosomes therefore represent an early endosomal compartment that is functionally distinct from vacuolar EEA1 endosomes.

In this context, it is noteworthy that ubiquitylation of EGFR, which is essential for allowing it to bind to ESCRT-0, serves two functions during EGFR trafficking (Eden et al., 2012). First, it helps to direct EGFR to the vacuolar early endosome, and thus prevents EGFR recycling to the plasma membrane. Second, it directs the sorting of EGFR to vesicles within the lumen of the vacuolar early endosome, thereby generating the MVB. Hence, the peripheral pool of ESCRT-0 might be important for the initial capture of ubiquitylated cargo that helps to control the balance between fast recycling pathways and transit to the vacuolar endosome. The failure of EGF to transfer efficiently to EEA1 endosomes when ESCRT-0 is depleted would be consistent with such an activity. Intriguingly, however, ESCRT-I also appears to be important for delivery of EGF to the vacuolar endosome, a role that might be linked to the more steady accumulation of ESCRT-I on early endosomes as these acquire EEA1. Taken together, these data point to the possibility that the delivery of cargo to the vacuolar endosome and its subsequent sorting might be mechanistically coupled, which could be important for the overall control of membrane flux through the pathway. Indeed, cargo separation within early endosomes has been shown to require factors, including EEA1, that promote early endosome fusion so as to couple endosome delivery with subsequent sorting events (Barysch et al., 2009).

We find that the peripheral ESCRT-0 endosomes are distinct from APPL1-positive endosomes, in line with the limited colocalisation observed between SNX15 and APPL1 (Danson et al., 2013). Our study is limited to fixed cell analysis, so we cannot exclude the possibility that EGF encounters Hrs after APPL1. Such a scenario seems highly unlikely given the very limited colocalisation between APPL1 and Hrs. Moreover, live-cell imaging of SNX15 and APPL1 demonstrates that the two markers behave independently with respect to EGF, with no evidence that SNX15 merges with or displaces APPL1 (Danson et al., 2013). Hence, although APPL1 endosomes provide an entry pathway to allow some EGFR to reach the EEA1-positive endosome (Zoncu et al., 2009), we believe that the ESCRT-0/SNX15 endosome represents an alternative and perhaps preferential route to the later compartment (see Fig. 8B) in the cell lines we have examined in detail (this study; Danson et al., 2013). These findings are in keeping with previous studies in HeLa cells, in which APPL1 endosomes were identified as a minor uptake pathway for EGF, with an important signalling role (Miaczynska et al., 2004). It is possible, however, that in some cells each acts to coordinate the entry of EGFR and other receptors from distinct plasma membrane domains. For example, a significant portion of EGFR is localised to the margins of COS7 cells (Leonard et al., 2008; Zoncu et al., 2009), and this pool of EGFR partitions mainly to APPL1 endosomes (Zoncu et al., 2009), a finding that we have confirmed.

The ability of both SNX15 (Danson et al., 2013) and ESCRT-0 (Raiborg et al., 2001; Stahelin et al., 2002) to interact with clathrin and PtdIns3*P* places these factors functionally close to each other and separate from APPL1. APPL1-positive endosomes mature into EEA1-positive endosomes through the WDFY2 compartment (Zoncu et al., 2009). It is possible that the ESCRT-0/SNX15 endosomes also acquire WDFY2, or undergo an equivalent switch before they acquire EEA1. Unfortunately, we have not been able to address the function of WDFY2 because we do not have antibodies to determine the localisation of endogenous WDFY2, and have been unable to obtain expression conditions under which GFP–WDFY2 does not aggregate in our chosen cell lines. We believe it likely, however, that ESCRT-0/SNX15 endosomes steadily acquire EEA1 over time, as they fuse with each other and move towards the cell centre. As endosomes enlarge, they contain higher concentrations of Rab5 (Rink et al., 2005) and PtdIns3*P* (Christoforidis et al., 1999; Zerial and McBride, 2001; Zoncu et al., 2009), both of which are important determinants for efficient targeting of EEA1 to the endosomal membrane (Stenmark et al., 1996; Patki et al., 1998; Simonsen et al., 1998).

SNX15 localises to a subpopulation of clathrin-coated pits and vesicles in addition to peripheral endosomes (Danson et al., 2013), suggesting that at least some of it associates with the membrane as the clathrin-coated vesicle is developing. Although a previous study has seen some clathrin-coated pits labelled with exogenously expressed Hrs (Mayers et al., 2013), we have failed to obtain evidence for such an early association of endogenous Hrs. This is in keeping with the increased colocalisation of EGF with Hrs between 2 and 5 min after ligand addition, suggesting that ESCRT-0 recruitment continues after coated-vesicle formation. These differences between the studies might reflect slightly aberrant behaviour of overexpressed Hrs, although equally we cannot exclude the possibility that any coated-pit-associated Hrs is unable to be accessed by antibodies. However, ESCRT-0 is not required for EGFR internalisation (Mayers et al., 2013), and is therefore not an obligate component of coated pits. Thus, the timing of ESCRT-0 recruitment to endocytic intermediates might be determined primarily by the rate of cargo ubiquitylation, with other factors (such as the presence of clathrin and adaptor proteins as well as PtdIns3*P* levels) also contributing to the recruitment pathway. Once ESCRT-0 is recruited to peripheral endocytic intermediates, it appears to exhibit vesicular transport role(s) upstream of and in addition to its established role in ILV formation, in securing efficient transit of cargo to the vacuolar endosome.

## MATERIALS AND METHODS

### Reagents

Rabbit anti-Hrs was raised using GST–Hrs as an immunogen and was generated by Eurogentec, Southampton, UK. The antibody was affinity-purified against GST–Hrs and adsorbed against GST. Sheep anti-VPS28 was as described previously (Bishop et al., 2002). Mouse anti-Hrs was from Enzo. Rabbit anti-EEA1 was from Cell Signaling Technology. Mouse anti-EEA1 was from BD Biosciences. Mouse anti-EGFR was Mab 108, purified from supernatants of the hybridoma clone HB-9764 (ATCC; Washington DC), grown in RPMI medium and 10% FCS under 5% CO_2_, and antibody affinity-purified using Protein-G–Sepharose. Rabbit anti-APPL1 and anti-CHMP4B were from Proteintech Europe (Manchester, UK). Rabbit anti-clathrin was from Abcam (Cambridge, UK). Fluorescent EGF was from Invitrogen (Paisley, Scotland) and secondary antibodies were from Jackson ImmunoResearch (West Grove, PA). Human SNX15 in pEGFP-C1 (Clontech) was a gift from Chris Danson and Pete Cullen, University of Bristol.

### Cell culture and transfection

HeLaM and A549 cells were grown in DMEM and 10% FCS under 8% CO_2_, and hTERT-RPE-1 cells were grown in the same medium supplemented with 10 µg/ml hygromycin B. DNA constructs were transfected using JetPEI (Qbiogene, Cambridge, UK) and analysed after 16–24 h. Transfection levels were optimised using pBlueScript as carrier DNA. For siRNA, cells were transfected using INTERFERin reagent (Qbiogene) and Dharmacon oligonucleotides.

### Western blotting

HeLaM cells grown in 12-well dishes were washed in PBS twice, then lysed in 50 µl of RIPA buffer per well containing Protease Inhibitor Cocktail III (Sigma-Aldrich, Poole, UK) and incubated on ice for 10 min. Cell lysates were centrifuged at 4°C and 14,000 ***g*** for 10 min to remove debris. Where appropriate, protein concentrations were measured using the bicinchoninic acid reagent (Thermo Fisher Scientific, Cramlington, UK), and equal amounts were loaded onto SDS-PAGE gels. Samples were transferred to PVDF membrane and blotted using IRDye 700CW- and 800C-labelled secondary antibodies from LI-COR Biosciences (Cambridge, UK).

### Immunofluorescence

Cells were fixed in 3% formaldehyde in PBS at room temperature, quenched using glycine and permeabilised in 0.05% SDS for 3 min. For experiments involving EEA1 dual labelling with CHMP4B or VPS28, cells were permeabilised with 0.1% Triton X-100. For experiments involving Hrs dual labelling with APPL1, CHMP4B or VPS28, cells were fixed in −20°C methanol, conditions that generated a slight nuclear background for anti-Hrs. For visualising endosomal pools of VPS28 or CHMP4B, cells were treated with 0.1% saponin in BRB80 buffer (80 mM PIPES-KOH, 1 mM MgCl_2_, 1 mM EGTA pH 6.8) for 5 min on ice before fixation. Cells were labelled for 30–60 min with primary antibody, washed and labelled with fluorescently labelled secondary antibodies from Jackson ImmunoResearch Laboratories (Stratech Scientific, Newmarket, UK). Fixed cells were imaged using a ×60 1.4 NA PlanApo objective on an Olympus IX70 microscope equipped for optical sectioning microscopy (Deltavision; Applied Precision, Issaquah, WA) and a CoolSnap HQ camera (Photometrics, Marlow, UK). Each *z*-series (0.2-µm intervals) was deconvolved and projected using SoftWorx (Applied Precision). In some cases, images were captured using an Olympus BX-60 microscope with a ×100 1.35 NA UPlanFl objective and a CoolSnap ES camera, using MetaVue. TIRF images were acquired using a Leica AM TIRF MC/DMI6000 B system. Briefly, images were acquired with a HC PL APO ×160 1.43 OIL CORR objective and an Andor iXon EM DU-897 back-illuminated EMCCD camera using the Leica Application Suite for Advance Fluorescence (LAS AF). All images were scaled using linear transformations in Adobe PhotoshopCS, MetaVue or ImageJ; PhotoshopCS and IllustratorCS were used to construct final figures.

### Automated colocalisation analysis

First, PolyParticleTracker (Rogers et al., 2007) was used to detect the precise locations of particles in each channel separately, after carefully choosing appropriate brightness and radius parameters to visually discriminate between true particles and noise. Then, each particle from channel 1 was considered in turn, and the distance to each particle in channel 2 was calculated. The nearest particles were then identified. If the distance between these particles was less than a threshold value the particles were considered as colocalised. This value was set at ∼200 nm. For still images taken with a ×60 objective and imaged with a CoolSnap camera, the precise distance of the threshold was two pixels (215 nm). For those taken with a ×100 objective, it was three pixels (193.5 nm). The degree of colocalisation of particles between channels was then taken as the percentage of channel 1 particles that are also observed in channel 2 or vice versa. To control for spurious colocalisation, the colocalisation routine was run again after the positions of particles were scrambled by adding a random number to the *x* and *y* coordinates. The colocalisation Matlab script (COLOCAL) and Graphical Unit Interface (GUI) are available from http://www.manchester.ac.uk/research/t.a.waigh/.

## Supplementary Material

Supplementary Material
