# Supplementary material for: ESCRT-0 marks an APPL1-independent transit route for EGFR between the cell surface and the EEA1-positive early endosome
Source: J Cell Sci. 2015 Feb 15;128(4):755–67. doi: 10.1242/jcs.161786 (PMC4327388; doi:10.1242/jcs.161786)
Supplement: Supplementary Material [file supp_128_4_755__index.html]

ESCRT-0 marks an APPL1-independent transit route for EGFR between the cell surface and the EEA1-positive early endosome — Supplementary Material 

# ESCRT-0 marks an APPL1-independent transit route for EGFR between the cell surface and the EEA1-positive early endosome

## JCS161786 Supplementary Material

**Files in this Data Supplement:**

- **Supplementary Material**
