# Supplementary material for: ESCRT-0 marks an APPL1-independent transit route for EGFR between the cell surface and the EEA1-positive early endosome
Source: J Cell Sci. 2015 Feb 15;128(4):755–67. doi: 10.1242/jcs.161786 (PMC4327388; doi:10.1242/jcs.161786)
Supplement: Supplementary Material [file supp_128.4.755_JCS161786.pdf]

Flores-Rodriguez et al.

Supplementary material

Supplementary Figure legends

Fig. S1. Controls for localisation of Hrs.

(**A**) HeLaM cell extracts were Western blotted for Hrs using mouse and rabbit anti-Hrs antibodies. (**B**) A549 cells were immunostained using rabbit anti-EEA1 and mouse anti-Hrs antibodies. (**C**) HeLaM cells were immunostained using rabbit anti-Hrs and mouse anti-Hrs antibodies. (**D**) HeLaM cells were immunostained using rabbit anti-EEA1 and mouse anti-EEA1 antibodies. (**E**) HeLaM cells were knocked down for ESCRT-0 components as indicated and stained with rabbit anti-Hrs and mouse anti-EEA1. Bars = 10 μm. Values show net percentages (i.e. scrambled values subtracted) of particles labelled by marker 1 also labelled by marker 2.

Fig. S2. Localisation of ESCRT markers

(**A**) **Distribution of EGF vs APPL1 and Hrs in COS7 cells.** Cos7 cells were pulse-chased with fluorescent EGF for 5 min total, then fixed and stained for APPL1 and Hrs. Scale bar = 10 μm. Insets magnified x3. Closed arrows show peripheral endosomes positive for both APPL1 and EGF. (**B**) Distribution of VPS28 in HeLaM cells. Control treated HeLaM cells (top) or cells depleted of TSG101 (bottom) were pulse-chased with fluorescent EGF as indicated and stained for IF. Scale bar = 10 μm.

Fig. S3. Distribution of CHMP4B in HeLaM cells.

(**A**) Control (top) or VPS4-depleted (bottom) HeLaM cells were stained for CHMP4B. (**B**) HeLaM cells were pulse-chased with fluorescent EGF for the indicated total times, permeabilised with saponin and fixed prior to IF. Scale bar = 10 μm. Insets magnified x3. Arrows indicate EGF endosomes co-labelled with CHMP4B.

Fig. S4. ESCRTs -0 and –I are required for EGF transit to EEA1 endosomes; independent siRNA oligos.

(**A**) HeLaM cells transfected with Hrs siRNA oligo 2 were pulse-chased with fluorescent EGF for the indicated total times and fixed for IF. (**B**) HeLaM cells transfected with Hrs siRNA oligo 1 were pulse-chased with fluorescent EGF (green) for 10 min, then fixed in formaldehyde. After incubating with or without Triton X-

100 cells were immunolabelled using an anti-EGFR antibody that recognises the extracellular domain (red). (**C**) Western blot of extracts from HeLaM cells transfected with control or UBAP1 siRNAs. (**D**) HeLaM cells transfected with UBAP1 siRNA oligo 2 were pulse-chased with fluorescent EGF for the indicated total times and fixed for IF. Scale bar = 10 μm.

Figure S1

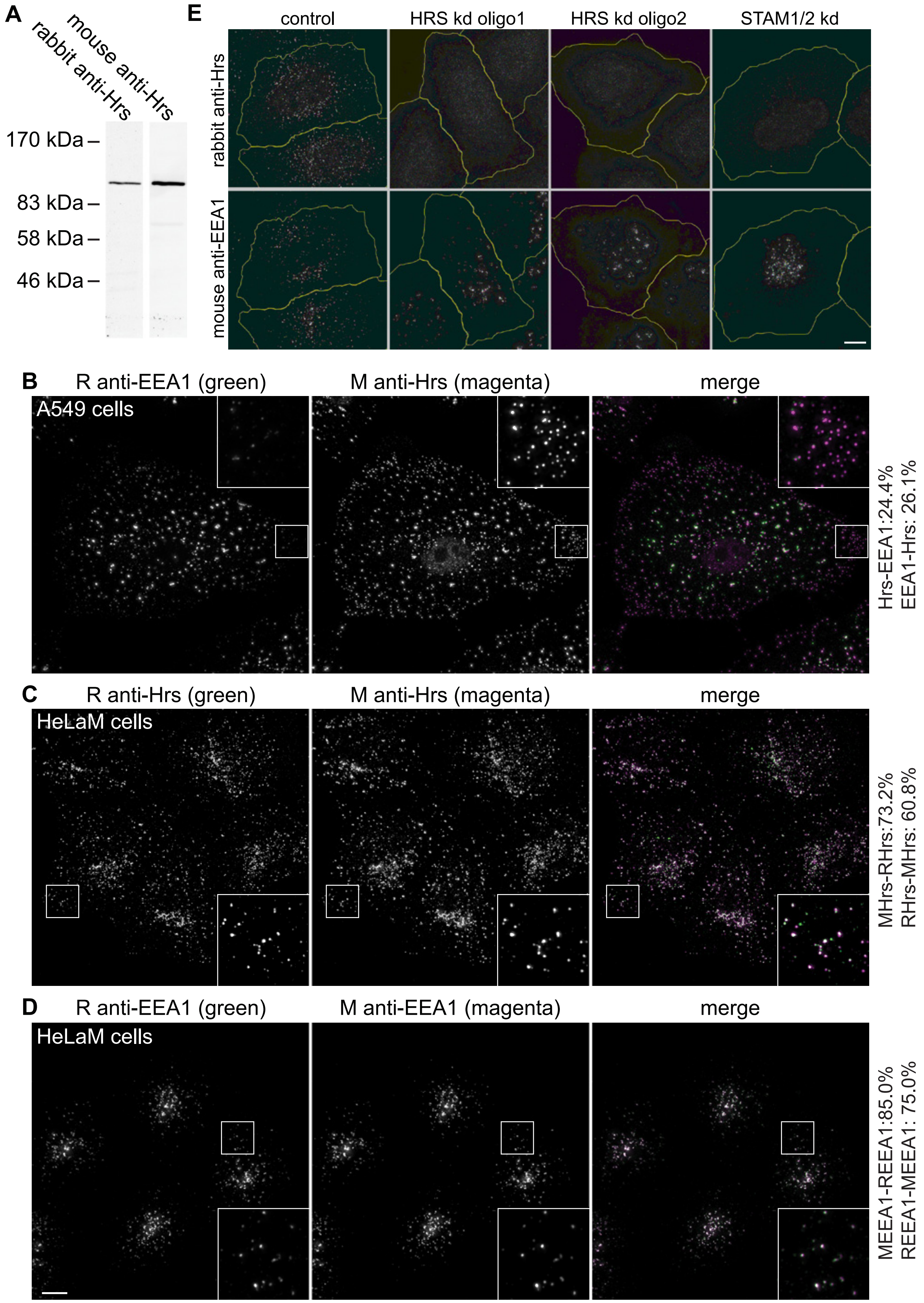
A
mouse anti-Hrs
rabbit anti-Hrs
170 kDa
83 kDa
58 kDa
46 kDa
E
control
HRS kd oligo1
HRS kd oligo2
STAM1/2 kd
rabbit anti-Hrs
mouse anti-EEA1
B
R anti-EEA1 (green)
M anti-Hrs (magenta)
merge
A549 cells
Hrs-EEA1:24.4%
EEA1-Hrs: 26.1%
C
R anti-Hrs (green)
M anti-Hrs (magenta)
merge
HeLaM cells
MHrs-RHrs:73.2%
RHrs-MHrs: 60.8%
D
R anti-EEA1 (green)
M anti-EEA1 (magenta)
merge
HeLaM cells
MEEA1-REEA1:85.0%
REEA1-MEEA1: 75.0%

Figure S2

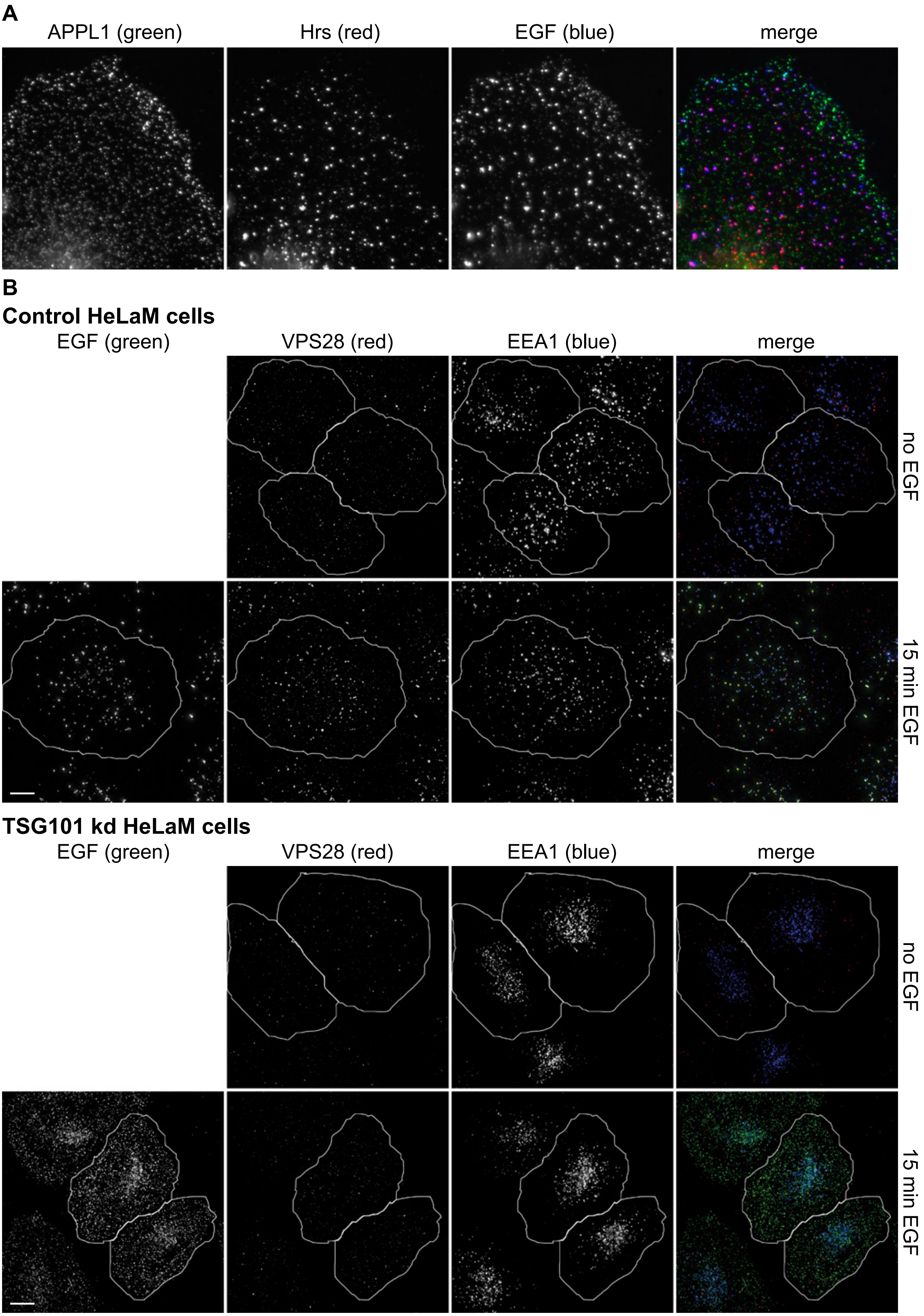
A
APPL1 (green)
Hrs (red)
EGF (blue)
merge
B
Control HeLaM cells
EGF (green)
VPS28 (red)
EEA1 (blue)
merge
no EGF
15 min EGF
TSG101 kd HeLaM cells
EGF (green)
VPS28 (red)
EEA1 (blue)
merge
no EGF
15 min EGF

Figure S3

A: HeLaM cells

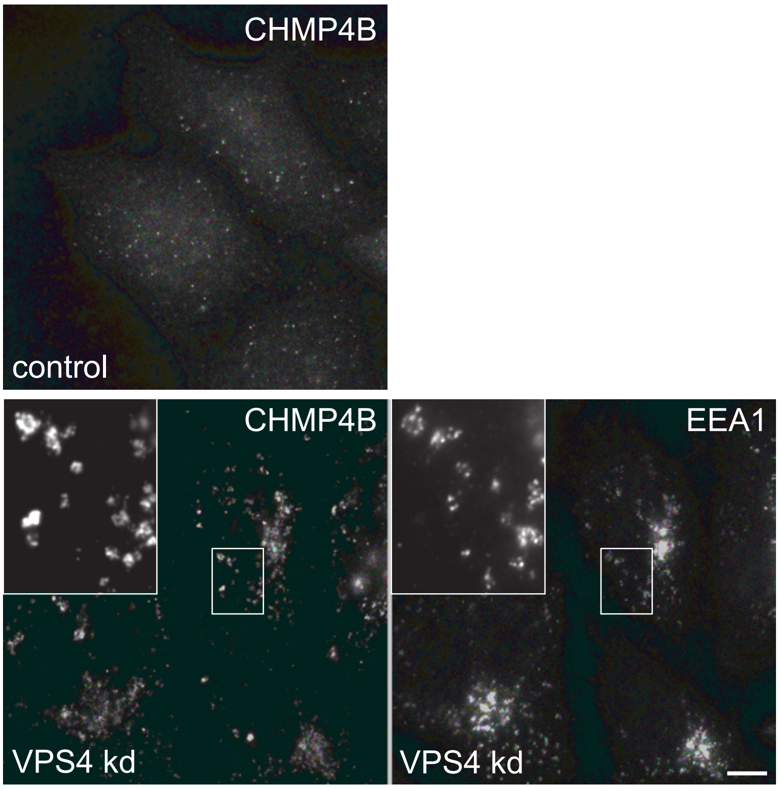

B: HeLaM cells

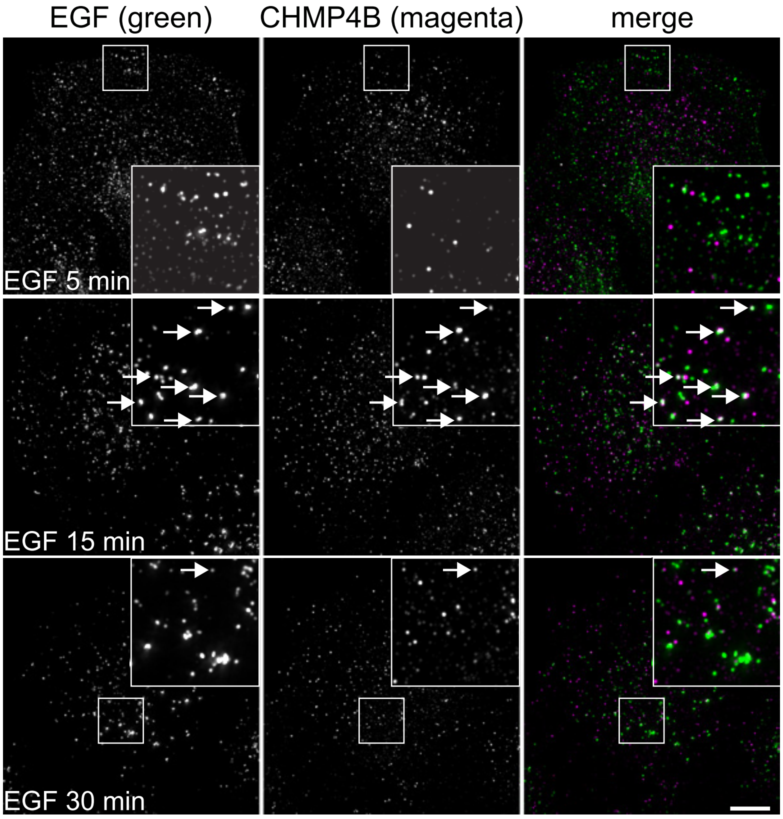

Figure S4

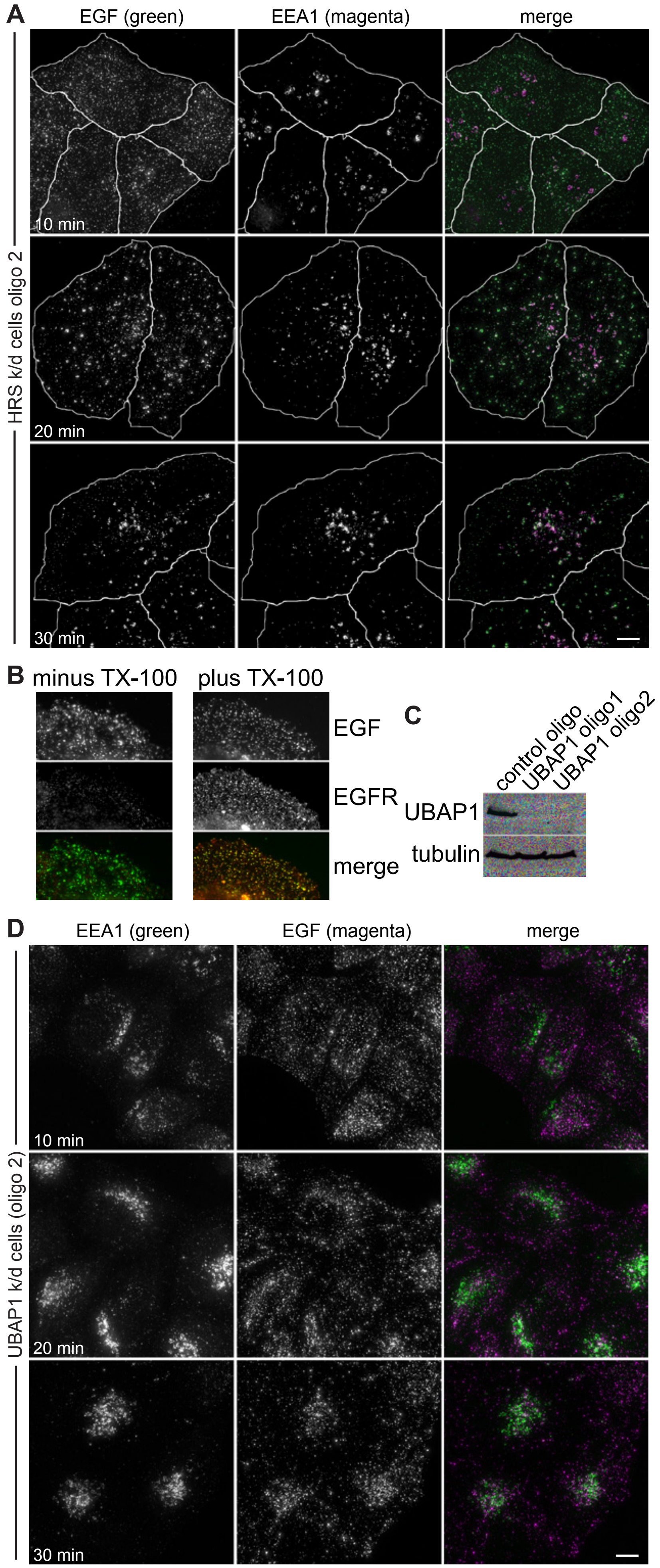
A
EGF (green)
EEA1 (magenta)
merge
HRS k/d cells oligo 2
10 min
20 min
30 min
B
minus TX-100
plus TX-100
EGF
EGFR
merge
C
control oligo
UBAP1 oligo1
UBAP1 oligo2
UBAP1
tubulin
D
EEA1 (green)
EGF (magenta)
merge
UBAP1 k/d cells (oligo 2)
10 min
20 min
30 min

Table S1. Quantification of endosomal marker colocalisation

Cell line	Channel 1	Channel 2	% Ch1 labelled in Ch2	Scramble % Ch1 labelled in Ch2	% Ch2 labelled in Ch1	Scramble % Ch2 labelled in Ch1	No of cells/ exps
**(A) Hrs vs EEA1**							
HeLaM	M EEA1	R EEA1	87.6 n=803	2.6	77.2 n=898	2.2	8/3
	M Hrs	R Hrs	78.9 n=2155	5.7	65.5 n=2631	4.7	12/3
HeLaM	R Hrs	M EEA1	9.9 n=13077	1.5	32.1 n=4028	5.0	48/2
	R EEA1	M Hrs	31.2 n=6938	3.8	14.1 n=13220	1.7	60/3
A549	M EEA1	R Hrs	40.6 n=996	3.1	26.2 n=1587	2.1	5/3
	M Hrs	R EEA1	25.8 n=1563	1.4	27.6 n=1468	1.5	4/3
RPE	R Hrs	M EEA1	13.2 n=1090	0.7	26.9 n=511	1.6	16/2
**(B) Hrs vs APPL1 (periphery)**							
HeLaM	M Hrs	R APPL1	7.9 n=1954	2.4	5.4 n=2800	1.7	9/2
**(C) Hrs vs CHC (periphery: TIRF)**							
HeLa	R CHC	M Hrs	1.9 n=2566	0.4	8.3 n=594	1.9	10/2
**(D) Hrs/EEA1 vs GFP-SNX15 (periphery)**							
RPE	M Hrs	GFP-SNX15	45.7 n=2069	2.8	73.5 n=1232	4.6	30/3
	M EEA1	GFP-SNX15	45.8 n=330	2.1	17.7 n=1191	0.6	29/3
**(E) ESCRT-1 (VPS28) vs EGF**							
RPE	EGF	Sheep VPS28	66.1 n=455	1.1	ND		12/2

Images were subjected to colocalisation analysis. The extent of colocalisation is expressed as the percentage of channel 1 labelled in channel 2, i.e. the number of channel 1 particles that were also positive in channel 2, and vice-versa, where n is the number of particles in the ‘template’ channel. ND; not determined. M; mouse, R; rabbit.
